# Supplementary material for: MetaRibo-Seq measures translation in microbiomes
Source: Nat Commun. 2020 Jun 29;11:3268. doi: 10.1038/s41467-020-17081-z (PMC7324362; doi:10.1038/s41467-020-17081-z)
Supplement: Supplementary file 10 — Supplementary Data 7 [file 41467_2020_17081_MOESM10_ESM.zip › File2/Confidence_High_Taxonomy/275841_out.krona.html]

Javascript must be enabled to view this page.

members
magnitude
magnitudeUnassigned
count
unassigned
taxon
rank

275841\_out

49

2
superkingdom
49

phylum
49
1239

49
class
186801

186802
order
49

1
family
186803

572511
1
genus

765821
1
species

SRS015431\_contig\_number\_57139

541000
family
48

1263
genus
4

species

SRS012273\_contig\_number\_22724SRS014412\_contig\_number\_17994SRS049773\_contig\_number\_31118SRS049896\_contig\_number\_33018
4
1262952

1898205
44
species

SRS011134\_contig\_number\_contig-100\_5077.183552SRS012849\_contig\_number\_5442SRS013098\_contig\_number\_30881SRS014235\_contig\_number\_contig-100\_708.267133SRS015578\_contig\_number\_12825SRS015782\_contig\_number\_36875SRS016335\_contig\_number\_6811SRS016629\_contig\_number\_1854SRS018575\_contig\_number\_contig-100\_4128.55966SRS018623\_contig\_number\_20677SRS018817\_contig\_number\_16329SRS018836\_contig\_number\_4217SRS019068\_contig\_number\_51806SRS019601\_contig\_number\_19798SRS020869\_contig\_number\_35577SRS023914\_contig\_number\_24465SRS024132\_contig\_number\_contig-100\_3079.75477SRS024435\_contig\_number\_contig-100\_1154.219346SRS042966\_contig\_number\_19108SRS047433\_contig\_number\_12937SRS049446\_contig\_number\_11472SRS049959\_contig\_number\_43002SRS050752\_contig\_number\_19230SRS050998\_contig\_number\_15122SRS053356\_contig\_number\_28739SRS056519\_contig\_number\_contig-100\_21706.21706SRS075878\_contig\_number\_17328SRS098571\_contig\_number\_18823SRS098881\_contig\_number\_contig-100\_25272.74803SRS1041032\_contig\_number\_15998SRS1041039\_contig\_number\_21743SRS1041157\_contig\_number\_1050SRS1055056\_contig\_number\_1954SRS143417\_contig\_number\_28894SRS143895\_contig\_number\_4032SRS144362\_contig\_number\_30851SRS144506\_contig\_number\_contig-100\_24230.77349SRS144537\_contig\_number\_25702SRS147557\_contig\_number\_29170SRS148196\_contig\_number\_8999SRS149325\_contig\_number\_4880SRS149784\_contig\_number\_13908SRS893279\_contig\_number\_16735SRS971275\_contig\_number\_2891
